# Supplementary material for: New Cases of Hypochromic Microcytic Anemia Due to Mutations in the SLC11A2 Gene and Functional Characterization of the G75R Mutation
Source: Int J Mol Sci. 2022 Apr 15;23(8):4406. doi: 10.3390/ijms23084406 (PMC9024435; doi:10.3390/ijms23084406)

Supplementary figures

New cases of hypochromic microcytic anemia due to mutations in the *SLC11A2* gene and functional characterization of the G75R mutation

Lídia Romero-Cortadellas [1*], Gonzalo Hernández[1,2*], Xènia Ferrer-Cortès[1,2], Laura Zalba-Jadraque[2], José Luis Fuster[3], Mar Bermúdez-Cortés[3], Ana María Galera-Miñarro[3], Santiago Pérez-Montero[2], Cristian Tornador[2] and Mayka Sánchez[1,2]**

[1] Universitat Internacional de Catalunya (UIC), Department of Basic Sciences, Iron metabolism: Regulation and Diseases. Sant Cugat del Vallès, Spain.
[2] BloodGenetics S.L. Diagnostics in Inherited Blood Diseases. Esplugues de Llobregat, Spain.
[3] Pediatric OncoHematology Service, Clinic University Hospital Virgen de la Arrixaca, Instituto Murciano de Investigación Biosanitaria (IMIB), Murcia, Spain.

* These authors contributed equally to this work

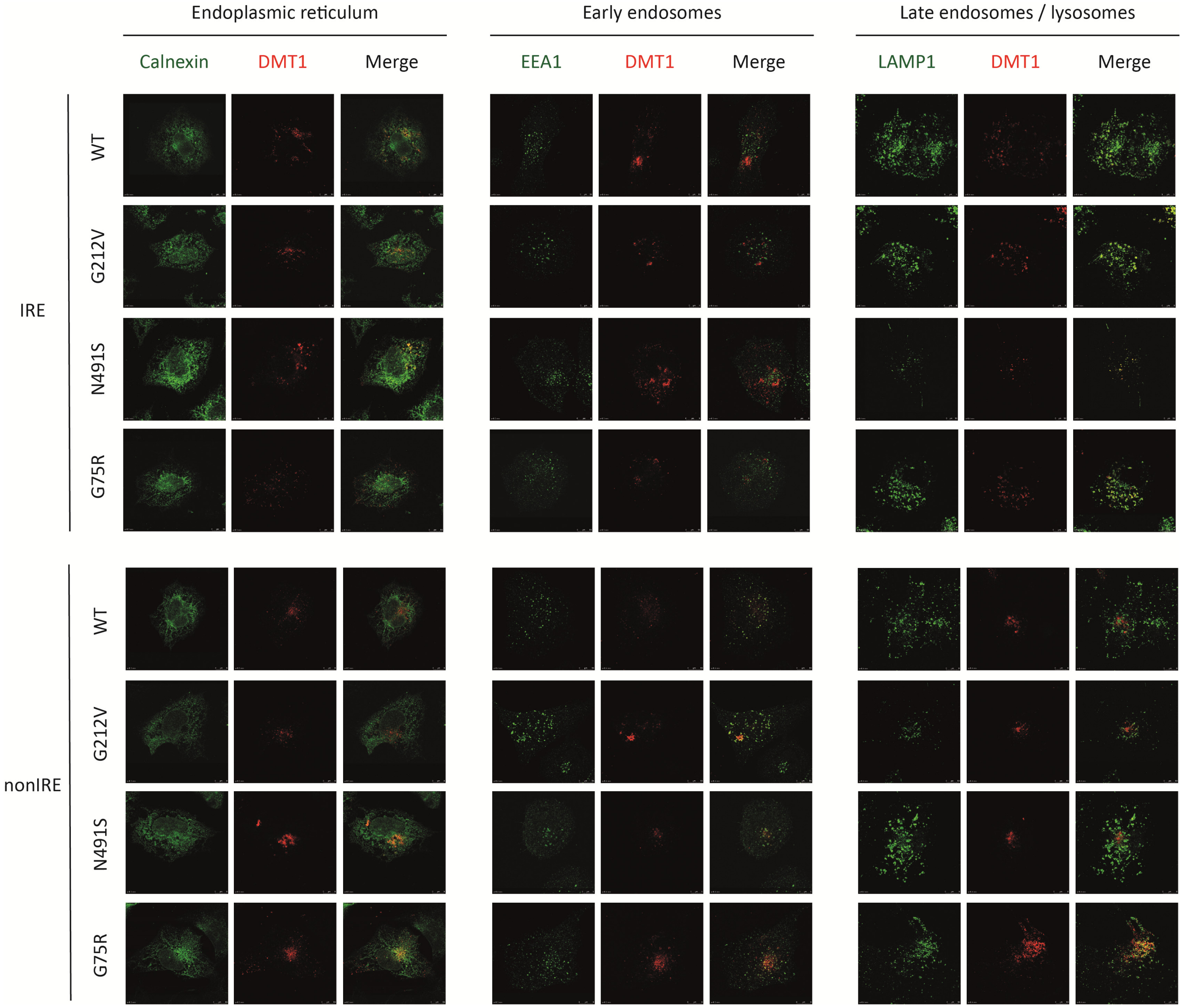

Supplementary Figure S1. Subcellular localization of wild-type and mutated DMT1 isoforms. Hutu-80 cells were transiently transfected with a pdsRed2-C1 construct with wild-type (WT), G212V, N491S and G75R mutants of -IRE and -nonIRE isoforms. Cells were incubated with Alexa 488-tagged primary antibodies: anti-calnexin to label endoplasmic reticulum and rabbit anti-LAMP1 to label late endosomes/lysosomes. The figure shows representative split and merged images for each experiment. IRE: iron-responsive element.

Supplementary Figure S2. Comparison of –IRE and –nonIRE DMT1 isoforms' subcellular localization. Co-localization is expressed as the percentage of DMT1 in the corresponding organelle (n=50). For the WT and each DMT1 mutation (G212V, N491S, G75R), co-localization of the -IRE versus the -nonIRE form in the endoplasmic reticulum (ER; A), early endosomes (B) and late endosomes/lysosomes (C) is compared. p<0.05 was considered significant. ****p < 0.0001.

A

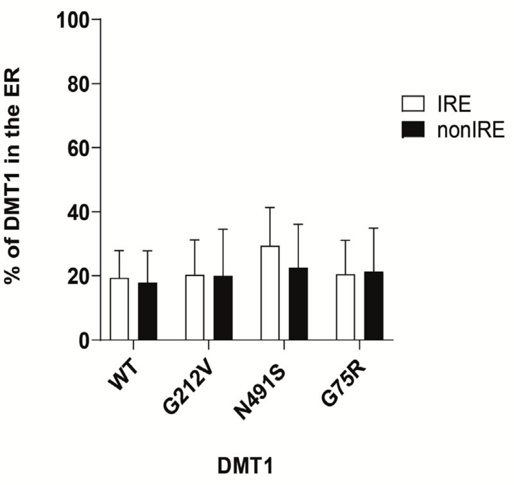

B

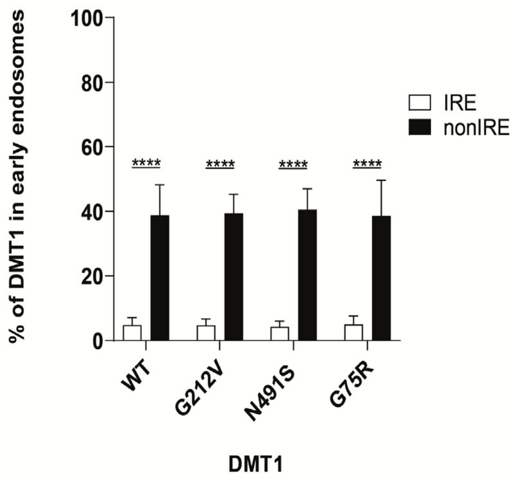

C

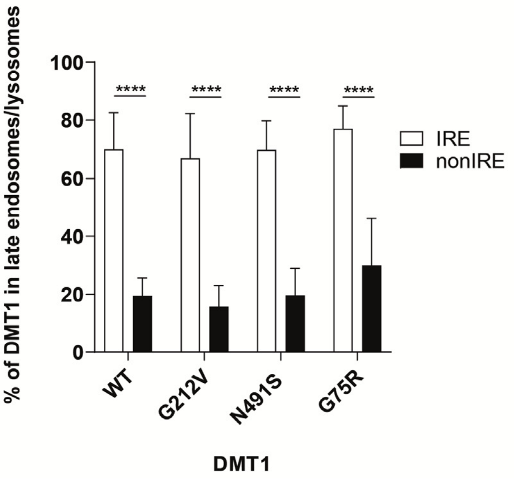

Supplement: Supplementary file 1 [file ijms-23-04406-s001.zip › ijms-1661857 supplementary.pdf]
